# Tumor growth rate as an intermediate trial endpoint

**DOI:** 10.1093/oncolo/oyag149

**Published:** 2026-04-17

**Authors:** Gregory Goldmacher

**Affiliations:** Clinical Imaging & Pathology, Merck & Co., Inc, Rahway, NJ, 07065, United States

The recently published analysis by Malinou et al.^1^ examining the association between model-derived tumor growth rate (g), overall survival (OS), and progression-free survival (PFS) in patients with metastatic non-small cell lung cancer (NSCLC) is a tremendously important contribution to the field. From the perspective of a radiologist working in pharmaceutical drug development, traditional imaging-based trial endpoints such as objective response rate (ORR) and PFS are critical in late-phase trials because of their widespread use and known acceptability to regulatory authorities. In early clinical development, efficacy assessment is typically based on ORR, supplemented with response duration and maximal reduction in the sum of diameters of a small sample of lesions (target lesions as defined by RECIST 1.1).

Unfortunately, the association between ORR and OS is weak in small cohorts, and cancer drug development history is replete with examples of drug candidates that looked promising in phase 1b or 2 based on ORR, then failed to improve survival in later trials. Thus, consequential decisions (dose selection and go/no-go to a later phase) must be made with inadequate inputs. Any opportunity to use intermediate endpoints that have a stronger association with OS is welcome. Prior research (eg, Bruno 2023)^2^ has shown g to be a promising intermediate endpoint at smaller cohort sizes, and there is an obvious biological rationale for using the growth of that component of tumor burden, which is growing during treatment, as a survival predictor. The publication of this paper by authors from the FDA sends a positive signal to the risk-averse pharmaceutical industry that this novel metric is worth exploring, and the large amount of data included in the analysis increases the credibility of the result.

There are, however, several considerations that are not addressed in the paper. First of all, there is no direct comparison between g and RECIST-based metrics as survival predictors, which drug developers will need to see before they start to deploy g as a decision-making tool. Second, the authors report that values of g differed between mechanisms of action (not surprising, given that different mechanisms show characteristically different time courses of tumor burden change). This means that the interpretation of g has the greatest validity within a trial, such as when comparing a monotherapy to a combination, or comparing different doses of the same treatment, and less validity when comparing novel treatments to historical data from drugs with a different mechanism. This suggests that more randomized phase 2 trials may be needed in the future, as historical comparisons of g may be impossible or inaccurate.

In this analysis, the growth rate model used the sum of diameters from RECIST 1.1 target lesions as the input. This is likely to underestimate the true value of g. Choosing target lesions is a form of sampling (the tumor burden for each subject is sampled, rather than measured in its entirety). Sampling lesions inherently implies that the outcome of interest (survival) is driven by the behavior of the average lesion (sampling estimates the population average). This is not true. It is not the average lesion that kills a patient, but their “worst” (most aggressive and treatment-resistant lesion). If that lesion was not selected as target, the model is blind to it, no matter how mathematically sophisticated. Also, previous work (eg, Maitland 2020)^3^ has shown that volumetric measurements, when used as input for g-modeling, yield better correlation with survival impact than single diameters. Since the authors only had access to RECIST data rather than the original scans, this is an understandable inherent limitation of the current analysis, but future research should not follow this method just because that is what the FDA did in this paper.

For the practicing oncologist, there can be hope that, once g has been more thoroughly validated and its calculation automated in clinical systems, it could be used to detect treatment failure more quickly than current qualitative approaches. Long before that, if g is validated in prospective trials, it could become a terrific tool for drug developers to rapidly evaluate early clinical stage assets and get much-needed drugs to patients more quickly and efficiently, and I applaud the authors for supporting this important step in its development.
